# Central Precocious Puberty: Treatment with Triptorelin 11.25 mg

**DOI:** 10.1100/2012/583751

**Published:** 2012-05-03

**Authors:** Elena Chiocca, Eleonora Dati, Giampiero I. Baroncelli, Alessandra Cassio, Malgorzata Wasniewska, Fiorella Galluzzi, Silvia Einaudi, Marco Cappa, Gianni Russo, Silvano Bertelloni

**Affiliations:** ^1^Adolescent Medicine, I Pediatric Division, Department of Obstetrics, Gynecology and Pediatrics, Azienda Ospedaliero-Universitaria Pisana, 56126 Pisa, Italy; ^2^Department of Gynecology, Obstetrics and Pediatrics, University of Bologna, 40138 Bologna, Italy; ^3^Department of Pediatrics, University of Messina, 98124 Messina, Italy; ^4^Department of Paediatrics, University of Florence, 50100 Florence, Italy; ^5^Paediatric Endocrinology, Regina Margherita Children's Hospital, 10126 Turin, Italy; ^6^Endocrinology Unit, Department University-Hospital, Bambino Gesù Children's Hospital, Tor Vergata University, 00165 Rome, Italy; ^7^Pediatric and Adolescent Endocrinology, Department of Pediatrics, San Raffaele Scientific Institute, Vita-Salute S. Raffaele University, 20132 Milan, Italy

## Abstract

*Background*. Few data are available on quarterly 11.25 mg GnRH analog treatment in central precocious puberty (CPP). *Aim*. To assess the efficacy of triptorelin 11.25 mg in children with CPP. *Patients*. 17 patients (16 females) with CPP (7.9 ± 0.9 years) were treated with triptorelin 11.25 mg/90 days. *Methods*. Gonadotropins, basal-, and GnRH-stimulated peak, gonadal steroids, and pubertal signs were assessed at preinclusion and at inclusion visit, 3 months, 6 months, and 12 months of treatment. *Results*. At 3, 6, and 12 months, all patients had suppressed LH peak (<3 IU/L after GnRH stimulation), as well as prepubertal oestradiol levels. Mean LH peak values after GnRH test significantly decreased from 25.7 ± 16.5 IU/L at baseline to 0.9 ± 0.5 IU/L at M3 (*P* < 0.0001); they did not significantly changed at M6 and M12. *Conclusions*. Triptorelin 11.25 mg/90 days efficiently suppressed the pituitary-gonadal axis in children with CPP from first administration.

## 1. Introduction

Central precocious puberty (CPP) refers to the development of gonadotropin dependent onset of puberty before the age of 8 years in girls and 9 years in boys [1]. CPP is caused by the premature reactivation of the hypothalamic-pituitary-gonadal-axis [1]. Gonadotropin stimulation of the gonads induces the increase in sex steroid secretion that is responsible for the premature onset of somatic sexual characteristics and is associated with growth spurt and accelerated skeletal maturation that compromises adult height [1].

The GnRH analogs represent the treatment of choice for CPP [2]. These drugs suppress gonadotropin secretion through a desensitization and downregulation of GnRH receptors, leading to reduction of gonadal steroids to prepubertal levels [3].

The monthly depot formulations of GnRH analogs are the main formulations used in the medical treatment of CPP [1, 2]. It has been demonstrated that they provide a steady release of drug [3] and improve significantly the short as well as the long-term outcome of children affected by CPP without relevant short-term or long-term side effects [1, 2, 4].

Three months depot formulations of GnRH agonists are now available and they are currently used in adults [5]. Since CPP may require several years of treatment [2], the use of three-month formulations would increase the effectiveness and the compliance with the therapy, but data on the use of quarterly GnRH analogs in CPP remain scarce [6–10].

This study assessed the efficacy and the tolerability of quarterly triptorelin 11.25 mg in children with CPP.

## 2. Patients and Methods

### 2.1. Patients

Children presented to our departments with clinical signs of precocious puberty (*n* = 47) were considered for the study; 17 patients (16 girls and 1 boy, mean chronological age 7.9 ± 0.9 years) with idiopathic CPP met all the requested clinical and hormonal criteria (see below) and were recruited in the study. 

The clinical and hormonal criteria were onset of pubertal characteristics development before 8 years in girls and 9 years in boys, pubertal response of LH to standard GnRH test (LH peak ≥7 IU/L); difference between (Δ) bone age (BA) and chronological age (CA) >1 year, uterine length ≥36 mm at ultrasound measurement in girls, testosterone levels >0.5 ng/mL in boys, negative MR imaging of hypothalamus-pituitary region [11].

### 2.2. Study Protocol

The patients were treated with triptorelin 11.25 mg every 90 days. Pharmacokinetic studies in adults demonstrated that the quarterly formulation of 11.25 mg triptorelin is steadily released over 91-day period [5].

Each dose of the drug was administered in outpatient hospital setting by experienced nurses in paediatric endocrinology and the date of each administration was registered on the clinical record of each child. Patients were actively contacted some days before the injections to be sure on the respect of administration protocol.

The study protocol based on preinclusion (first visit and endocrine testing), inclusion (M0: visit and first triptorelin injection made within 1 month after pre-inclusion), and four treatment visits in the occurrence of drug injection (M3, M6, M9, and M12). At each visit, except M9 visit, pubertal stage and hormonal parameters (basal and stimulated gonadotropins, 17*β*-estradiol or testosterone) were assessed. Auxological parameters (height, weight, and growth velocity), bone age, and uterine length (in girls) were taken at M0, M6, and at M12 visits.

The primary endpoint of the trial was to assess the efficacy of triptorelin 11.25 mg in suppressing GnRH-stimulated LH peak at M3, M6, and M12. Optimal suppression was defined as a LH peak ≤3 IU/L.

Secondary endpoints included suppression to prepubertal levels of stimulated FSH after GnRH stimulation and basal gonadal steroids, as well as variations of clinical (height, height SD score, growth velocity, weight, and pubertal stage) and radiological parameters (bone age). Assessment of triptorelin plasma levels was performed at M3, M6, and M12 before the drug injection.

### 2.3. Consent

Written informed consent was obtained from both parents or by the liable parent or by the legal guardian, when applicable; the assent was also obtained by the children, when they were able to understand the explanation provided by the endocrine paediatrician.

The study was approved by the Ethics Committee for human investigations of the hospitals involved in the study.

### 2.4. Methods

Blood samples for measurements of biochemical parameters were obtained in fasting state between 08.00 and 09.00. LH, FSH (baseline and GnRH stimulated (100 *μ*g i.v.)), and 17*β* estradiol were assayed at time points M0, M3, M6, and M12.

17*β* estradiol was assayed by commercial RIA method (Estradiol US, Diagnostic System Laboratory, Webster TX USA) with threshold of sensitivity of 2.0 pg/mL, and inter- and intraassay coefficients of variation (CVs) were 9.4% and 7.5%, respectively. LH and FSH were assayed by commercial microparticle enzyme immunoassay method (AXsym Abbott LH and FSH, Abbott Park, USA) with threshold of sensitivity of 0.1 IU/L for both hormones. For LH, inter- and intraassay CVs were 7.3% and 5.1%, respectively. For FSH, inter- and intraassay CVs were 6.4% and 4.9%, respectively. All endocrine assays were carried out at in independent laboratory (Exacta Laboratory, Verona Italy) blinded to the treatment regimen.

Triptorelin residual plasma levels were assessed by RIA methods. The samples were collected at M3, M6, and M12 just before the injection.

Pubertal development was assessed according to Tanner and Whitehouse [12]. Standing heights were measured with a wall-mounted stadiometer. Heights were expressed as real measured value or as SDS. The height SD score for the chronological age was obtained from the tables of Tanner and Whitehouse [12]. Midparental heights were adjusted for the female ([father's height + mother's height]/2 − 6.5 cm) or male ([father's height + mother's height]/2 + 6.5 cm).

Bone age was determined using X-rays of the nondominant hand and wrist and estimated by the Greulich and Pyle method [13]. Predicted adult height was calculated according to the tables of Bayley and Pinneau [14].

Body mass index (BMI) was calculated according to the formula: weight (kg)/height (m^2^), and BMI SD score was calculated according to age and sex [15].

### 2.5. Statistical Analysis

Results are expressed as mean ± SD. Analysis of variance was used to compare the on-study results with baseline values. A “*P*” value less than 0.05 was considered to be significant in all instances. Statistical analyses were carried out using the SPSS for Windows software program.

## 3. Results

### 3.1. Clinical and Radiological Features at Inclusion and during Followup

Clinical features are reported in Table 1. Height increased during the study period, but growth velocity (cm/year) decreased to prepubertal values. Mean bone age progressed 11 months, predicted adult height increased, and resulted close to midparental height at M12 (Table 1). The BMI, as absolute values or SDS, unchanged (Table 1). Breast development (B stage) as well as uterus length decreased from M0 (2.7 ± 0.8, 45.7 ± 7.8 mm; resp.) to M12 [2.1 ± 0.8 (*P* = 0.0001 versus M0), 35.3 ± 5.1 (*P* = 0.0002 versus M0), resp.]. Testicular volume did not change in the single boy.

### 3.2. LH, FSH, and Gonadal Steroids at Baseline and during Treatment

Endocrine data at inclusion and during followup are reported in Table 2. A significant decrease of both basal and peak LH values was found from baseline to M3 (*P* < 0.0001); thereafter the hormone values did not significantly changes (Table 2).

All the patients (100%) showed LH peaks at M3, M6, and M12 below the cutoff for optimal suppression (Table 2). All the girls (100%) have suppressed levels of estradiol (<70 pmol/L) at M3, M6, and M12 (Table 2). Testosterone in the single boy decreased from pubertal (baseline: 12.8 nmol/L) to prepubertal values during followup (M12: 0.3 nmol/L).

Basal and peak FSH levels decreased significantly at M3 (*P* < 0.0001) compared to the start of treatment and did not changed thereafter (Table 2).

### 3.3. Residual Plasma Levels of Triptorelin

Serum triptorelin levels were detectable in all the patients during followup (M3: 84.0 ± 1.0 pg/mL; M6: 64.0 ± 5.0 pg/mL; M12: 42.0 ± 2.0 pg/mL).

### 3.4. Tolerance

The drug was well tolerated. No patient withdrew from the study because of adverse events. The possible related side effects during treatment were headache (22%) and flushes (1%). Three girls reported light vaginal bleeding for a total of 4 events. No local reaction at injection site was observed.

## 4. Discussion

GnRH analogs are the treatment of choice for CPP [2]. At the present, monthly depot preparations are usually employed, at least in Europe [1, 2].

Quarterly and yearly depot formulations of GnRH analogs are now available, having potential advantages in improving compliance and the quality of life of children under treatment [6–8, 16–20].

The efficacy of quarterly preparations in adult clinical setting has been well documented [5, 21, 22], but the studies evaluating these new formulations in CPP remain scarce [6–10, 16–18]. Thus, we assessed triptorelin 11.25 mg administered every 90 days in suppressing gonadotropin secretion in patients with CPP.

In this study, triptorelin 11.25 mg significantly reduced LH values at M3 in comparison with baseline levels and all peaks were below the chosen cutoff value from first treatment cycle. Optimal LH suppression was maintained up to M12 without significant variations during followup, despite residual triptorelin levels reduced below the reported therapeutic range [3], suggesting that even low serum levels of the agonist is able to desensitize LH secretion. FSH and gonadal steroids levels also decreased to prepubertal values, confirming the suppression of pituitary-gonadal axis. Clinical and imaging data were in agreement with a drug-induced prepubertal hormonal milieu, but longer followup is needed to give sound conclusions on the effectiveness of improving adult height.

On biochemical point of view, our data were better than those reported in previous studies with triptorelin 11.25. Martínez-Aguayo et al. [8] treated 19 patients with this formulation and found an adequate LH suppression in 90% at 3-month of treatment. The 100% of suppression was achieved only from 6 to 24 months [8]. Carel et al. [7] tested quarterly triptorelin in 52 girls with CPP; 85% showed LH peak below their cutoff value (3 IU/L) after the first drug dose and 97% after the second dose. The better suppression of LH peak we found from the first drug administration may be due to the strict protocol rules and the administrations made by experienced nurses. At this regard, a subset of Carel's population, which excluded patients with major protocol violations, was fully suppressed at both 3 and 6 months of therapy [7]. In the present study, the 100% triptorelin efficacy would be confirmed even considering a very restrictive value of 2 IU/L as cutoff for LH peak after GnRH stimulation test.

Regarding the other quarterly GnRH analogs, Carel et al. [6] evaluated leuprorelin 11.25 mg in a group of 44 girls: 93% of the patients had suppressed LH peak (cutoff value 3 IU/L) after 3 months and 98% after 6 months of therapy. Nevertheless, the most appropriate dose of leuprorelin to treat CPP remains a debated issue. Badaru et al. [9] compared leuprorelin 3.75 mg and 7.5 mg monthly to the 11.25 mg/3 months. They found higher stimulated LH and FSH values with the 3.75 mg/monthly and 11.25 mg/3 months compared with 7.5 mg/month, suggesting that the latter compound was more effective in suppressing the gonadotropins, although no difference in sex steroid levels were found [9]. Mericq et al. [10] compared, in an open 1-year study, three different doses of leuprorelin: 7.5 mg/monthly, 11.25/3 months, and 22.5/3 months. LH was suppressed in all the patients on 22.5 mg/3 months, in all patients on 7.5/mg monthly by 9 months, and in all patients on 11.25 mg by 12 months. Recently, Fuld et al. [23] compared monthly (7.5 mg) and quarterly (11.25 mg and 22.5 mg) depot formulations of leuprolide acetate in the treatment of CPP. Mean stimulated LH and FSH levels during treatment were higher in the low-dose 11.25-mg 3-month group and more LH levels above cutoff (4 IU/L) were observed in comparison with the other two dose groups. However, no differences in estradiol levels, growth velocity, or bone age progression were observed in the three groups [23]. The authors concluded that all leuprolide acetate formulations determined prompt and effective suppression of puberty, but higher dosing may be required for multi-monthly preparation in some circumstances [23]. Albeit definitive conclusions about the effectiveness of different leuprorelin doses in CPP require additional comparative studies and long-term evaluation, the lesser suppression reported in patients treated with lower doses [9, 23, 24] may indicate that the less biological activity of leuprorelin [3] may possibly worsen long-term outcome when administered at the minimal doses [9, 24]. At this regard, Massart et al. [25] reported that final height was significantly lower of about 0.6 SDS in girls treated with monthly leuprorelin 3.75 mg in comparison with those treated with monthly triptorelin 3.75 mg (*P* < 0.05).

Regarding goserelin, Isaac et al. [18] compared the 10.8 mg formulation administered every 9–12 weeks with that of 3.6 mg given monthly over a 12-month period. They found that 71% of the patients treated with quarterly formulations and 44% of those treated with monthly formulation had inadequate clinical and/or biological suppression [18]. They concluded that patients treated with goserelin depot may require an increase in injection frequency, especially those treated with quarterly formulation [18]. Trueman et al. [17] also assessed quarterly goserelin 10.8 mg in CPP and found that 2% of their patients had inadequate suppression at 8-week followup. The percentage rose to 13% at 12 weeks, leading to the conclusion that some patients treated with goserelin may require increased injection frequency to obtain adequate suppression [17].

Recently, very long delivery system was used to treat CPP in USA, as subcutaneous histrelin implants (50 mg/year, releasing 60 *μ*g/daily) [19, 20]. Two-year followup demonstrated effectiveness and safety of this formulation, but larger samples and long-term outcome should be evaluated [19, 20].

Overall, the new formulations of GnRH analogs were well tolerated, as we found in the present report. Sterile abscess formation, reported as a complication of leuprolide acetate (15 mg monthly) and histrelin acetate (50-mg yearly) [26], has been not observed with triptorelin depot in this and other reports [7, 8, 16, 26].

In conclusion, the present study showed that quarterly triptorelin 11.25 mg is effective in suppressing pituitary-gonadal axis during the first year of treatment, when strict rules of administration are warranted. Comparing our results with published data (Table 3), the efficacy of triptorelin 11.25 mg seems to be better from first treatment cycle than that of other quarterly GnRH analogs administered at similar doses (Table 3). Direct short- and long-term comparative trials among the various GnRH analog and different formulations using homogeneous criteria to define cutoff level for adequate suppression would be performed to give better indications for clinical practice. In addition, more information on the relationship between on-treatment measures of gonadotropin suppression and final outcomes is needed [2].

## Figures and Tables

**Table 1:** Clinical data at the beginning of GnRH analog treatment (M0) and after 12 months of quarterly triptorelin 11.25 mg (M12).

	M0	M12
Bone age, years	9.8 ± 1.2	10.7 ± 1.1
Height, cm	132.7 ± 7.8	139.1 ± 8.6°
Height, SDS*	−0.4 ± 1.3	−0.3 ± 0.9
Growth velocity, cm/year	11.0 ± 4.9	4.9 ± 2.1°°
Predicted adult height, cm	156.3 ± 9.6	160.8 ± 6.5
Predicted adult Height, SDS	−0.7 ± 1.5	−0.2 ± 1.1
Body mass index, kg/m^2^	18.0 ± 2.5	18.6 ± 2.4
Body mass index, SDS	2.8 ± 2.7	2.7 ± 2.0
Midparental height, cm	161.0 ± 4.5	
Midparental height, SDS	−0.2 ± 0.8	

*SDS for bone age; °*P* < 0.02 versus M0; °°*P* < 0.001 versus M0.

**Table 2:** Biochemical data at baseline and 3, 6, and 12 months of quarterly triptorelin 11.25 mg.

	baseline	3 months	6 months	12 months
Basal LH, IU/L	1.9 ± 1.6	0.3 ± 0.2	0.4 ± 0.4	0.4 ± 0.2
Peak LH, IU/L	25.7 ± 16.5	0.9 ± 0.5	1.0 ± 0.5	1.0 ± 0.5
Peak LH < 3 IU/L	—	17/17	17/17	17/17
Basal FSH, IU/L	4.1 ± 2.2	0.9 ± 0.5	1.1 ± 0.5	1.5 ± 0.8
Peak FSH, IU/L	11.7 ± 4.7	1.3 ± 0.6	1.3 ± 0.6	1.9 ± 0.9
17*β* estradiol, pmol/L°	58.0 ± 45.5	30.8 ± 7.7	27.8 ± 12.1	34.1 ± 12.1
17*β* estradiol < 70 pmol/L°	11/16	16/16	16/16	16/16

°data related to the 16 girls enrolled in the study. °Peak < 2 IU/L 17/47 a M3, M6, M12.

**Table 3:** Efficacy of LH peak suppression of long-acting GnRH analogs in CPP (short- and medium-term studies in *de novo*-treated children).

		Sex			Patients with optimal LH suppression, %			
Author	GnRH analog	F, *n*	M, *n*	Cutoff LH peak, IU/L	3 months	6 months	9 months	12 months
Carel et al. [6]	Leuprorelin 11.25 mg	40	4	3.0	93.0	98.0	—	—
Meriq et al. [16]	Leuprorelin 11.25 mg	10	1	3.0	—	—	—	88°
Carel et al. [7]	Triptorelin 11.25 mg	54	10	3.0	85.0(97^*∧*^)	97(97^*∧*^)	—	95(97^*∧*^)
Martínez-Aguayo et al. [8]	Triptorelin 11.25 mg	19	1	3.0	90.0	100	100	100^§^
Trueman et al. [17]	Goserelin 10.8 mg	23	6	1.7	67.0	—	—	—
Isaac et al. [18]	Goserelin 10.8 mg	23	5	2.0	—	—	—	81*
Lewis and Eugster [19]	Histrelin 50-mg	20	—	4.0	100	100	100	100°°
Present study	Triptorelin 11.25 mg	16	—	3.0	100^*∧∧*^	100^*∧∧*^	100^*∧∧*^	100^*∧∧*^

°data at 18 months of leuprorelin administration.

^*∧*^data in peer-protocol population (*n* = 37 patients who had received all doses of triptorelin 11.25 and without no major protocol violation).

^§^LH peak below 3.0 IU/L was documented at 15, 18, and 21 months of followup. At month 24, 1 patient showed a leak peak at 3.0 IU/L.

*20/28 (71%) required treatment injections at 6–10 weekly intervals.

°°data were confirmed in the 12–24 months of followup [20].

^*∧∧*^using the lower cutoff value of 2.0 IU/L [18], the percentage of patients with optimal suppression did not change.
